# A multi-level implementation strategy to increase adoption of chiropractic care for low back pain in primary care clinics: a randomized stepped-wedge pilot study protocol

**DOI:** 10.1186/s12998-024-00565-w

**Published:** 2025-02-20

**Authors:** Eric J. Roseen, André Bussières, Rocky Reichman, Celia Bora, Jennifer Trieu, Kirsten Austad, Charles Williams, Ryan A. Fischer, Danielle Parrilla, Lance D. Laird, Michael LaValley, Roni L. Evans, Robert B. Saper, Natalia E. Morone

**Affiliations:** 1 Section of General Internal Medicine, Department of Medicine, Boston University Chobanian & Avedisian School of Medicine and Boston Medical Center, Boston, MA USA; 2 School of Physical and Occupational Therapy, Faculty of Medicine and Health Sciences, McGill University, Montreal, Québec Canada; 3 Département Chiropratique, Université du Québec à Trois-Rivières, Trois-Rivières, Québec Canada; 4 NeighborHealth, Boston, MA USA; 5 Department of Family Medicine, Boston University Chobanian & Avedisian School of Medicine and Boston Medical Center, Boston, MA USA; 6 Greater Roslindale Medical and Dental Center, Boston, MA USA; 7 Evans Center for Implementation and Improvement Sciences, Boston University, Boston, MA USA; 8 Department of Veterans Affairs Bedford, Bedford, MA USA; 9 Department of Biostatistics, Boston University School of Public Health, Boston, MA USA; 10 Integrative Health & Wellbeing Research Program, Earl E. Bakken Center for Spirituality and Healing, School of Nursing, University of Minnesota, Minneapolis, MN USA; 11 Department of Wellness and Preventive Medicine, Cleveland Clinic, Cleveland, OH USA

**Keywords:** Chiropractic care, Low back pain, Primary care, Community health center, Nonpharmacologic treatment, Chronic pain

## Abstract

**Introduction:**

Limited adoption of first line treatments for low back pain (LBP) in primary care settings may contribute to an overreliance on pain medications by primary care providers (PCPs). While chiropractic care typically includes recommended nonpharmacologic approaches (e.g., manual therapy, exercise instruction, advice on self-care), implementation strategies to increase adoption of chiropractic care for LBP in primary care clinics are understudied, particularly in underserved communities.

**Methods:**

We will use a stepped-wedge cluster randomized controlled pilot trial design to evaluate the feasibility of a multi-level implementation strategy to increase adoption of chiropractic care for LBP in primary care clinics at community health centers. Key barriers and facilitators identified by site champions and other key stakeholders will help us to develop and tailor implementation strategies including educational materials and meetings, developing a network of local chiropractors, and modifying the electronic health record to facilitate referrals. Three primary care clinics will be randomized to receive the implementation strategy first, second, or third over a fourteen-month study period. At our first clinic, we will have a four-month pre-implementation period, a two-month implementation deployment period, and a subsequent eight-month follow-up period. We will stagger the start of our implementation strategy, beginning in a new clinic every two months. We will evaluate the proportion of patients with LBP who receive a referral to chiropractic care in the first 21 days after their index visit with PCP. We will also evaluate adoption of other guideline concordant care (e.g., other nonpharmacologic treatments) and non-guideline concordant care (e.g., opioids, imaging) over the study period.

**Discussion:**

LBP is currently the leading cause of disability worldwide. While there are several treatment options available for individuals with LBP, patients in underserved populations do not often access recommended nonpharmacologic treatment options such as chiropractic care. The results from this study will inform the development of practical implementation strategies that may improve access to chiropractic care for LBP in the primary care context. Furthermore, results may also inform policy changes needed to expand access to chiropractic care in underserved communities.

**Clintrials.gov NCT#:**

NCT06104605.

**Supplementary Information:**

The online version contains supplementary material available at 10.1186/s12998-024-00565-w.

## Background

Low back pain (LBP) is the leading cause of disability and healthcare costs in the United States [1–3]. At least four of five adults will experience LBP in their lifetime and it is among the most common conditions managed by primary care providers (PCPs) [4–6]. Current clinical practice guidelines for the management of LBP in primary care emphasize the initial use of nonpharmacologic treatments (e.g., acupuncture, spinal manipulation, massage) as first line therapy for acute or chronic LBP with or without leg pain [7–10]. However, adoption of these recommendations by PCPs can be challenging as nonpharmacologic treatments are often not available in primary care clinics and communication may be limited between PCPs and community-based nonpharmacologic treatment providers (e.g., acupuncturists, chiropractors, massage therapists) [11]. Low adoption of nonpharmacologic treatments for LBP in primary care settings may contribute to reliance on pain medications such as non-steroidal anti-inflammatory drugs (NSAIDs) or opioids [12, 13]. Use of common recommended approaches, such as chiropractic care, is lowest among racial and ethnic minoritized groups, and in low-income and federally-insured populations [14]. This study will evaluate the feasibility of a multi-level implementation strategy to increase adoption of chiropractic care for LBP in primary care clinics that serve under-resourced communities.

Typical components of chiropractic care for LBP include patient education, advice on self-care, exercise instruction, and manual therapy [15]. Thus, chiropractic care incorporates advice to remain physically active and self-care strategies, which are endorsed as part of initial care for LBP across all major guidelines [7, 9, 10, 16, 17]. Spinal manipulation, a type of manual therapy commonly provided as part of chiropractic care for LBP, has been shown to improve pain and function outcomes, which can reduce pain interference with normal activities and support patient engagement in self-care. Spinal manipulation for LBP is supported by systematic reviews and meta-analyses of large randomized controlled trials [18–20] and clinical practice guidelines [7, 9, 10, 16, 17]. It is safe [21, 22] and cost-effective [23, 24]. A prior large multi-site pragmatic trial found combined chiropractic care and usual care to be more effective than usual care alone for LBP [25]. Furthermore, adults who initially access chiropractic care for LBP, compared to those who seek care first with their PCP, are less than half as likely to receive opioids in the short or long-term [26–28]. Thus, improved collaboration between PCPs and the 70,000 currently practicing US-based Doctors of Chiropractic (DCs) is a promising approach in the effort to de-emphasize opioids and other pain medications and emphasize nonpharmacologic management for LBP.

Adoption is defined as the intention, initial decision, or action to try or employ an innovation or evidence-based practice [29]. Referrals from PCPs to DCs is a measurable form of adoption of chiropractic care in primary care settings. Despite strong evidence and guidelines supporting use of chiropractic care for LBP, referrals to DCs are rare [12, 30]. Initiating or engaging in conversations with patients about chiropractic care for LBP without placing a referral, which may not be required by insurers, may also signal PCP adoption [31]. Our multi-level implementation strategy will target several known barriers that may explain limited adoption of chiropractic care in primary care settings. First, PCPs may have limited opportunity to learn about chiropractic care or meet DCs while in medical training [32]. Second, less than 10% of DCs work in hospitals or other conventional medical settings and PCPs may have few opportunities to engage with DCs while in practice [32, 33]. With the absence of DCs in large healthcare delivery systems, collaborative relationships may rely on limited networks of communication between PCPs and community-based DCs [11, 32]. Third, there are few tools available or policies in place that would facilitate referrals to chiropractic care or foster collaborative relationships between DCs and PCPs. Efforts to make referrals easier, such as shared decision-making tools or sample language to describe chiropractic care, could facilitate adoption. Development and testing of these implementation strategies is needed to increase adoption of chiropractic care for LBP in primary care settings.

To address identified knowledge gaps, we present the design and methods for a pilot trial evaluating the feasibility of a multi-level implementation strategy aimed at improving the adoption of chiropractic care for patients with LBP in primary care clinics at three community health centers (CHCs) in eastern Massachusetts. The implementation strategy will be tailored to each primary care clinic through stakeholder engagement (i.e., site visits, interviews). We will assess the feasibility of measuring adoption of chiropractic care, our anticipated primary outcome for a future large fully powered cluster-randomized trial of implementation strategies to increase adoption of chiropractic care. We will also assess whether it is feasible to measure adoption of other guideline concordant care (e.g., other nonpharmacologic treatments) and non-guideline concordant care (e.g., prescribed pain medications) over the study period.

## Methods

### Study design

**Fig. 1:**
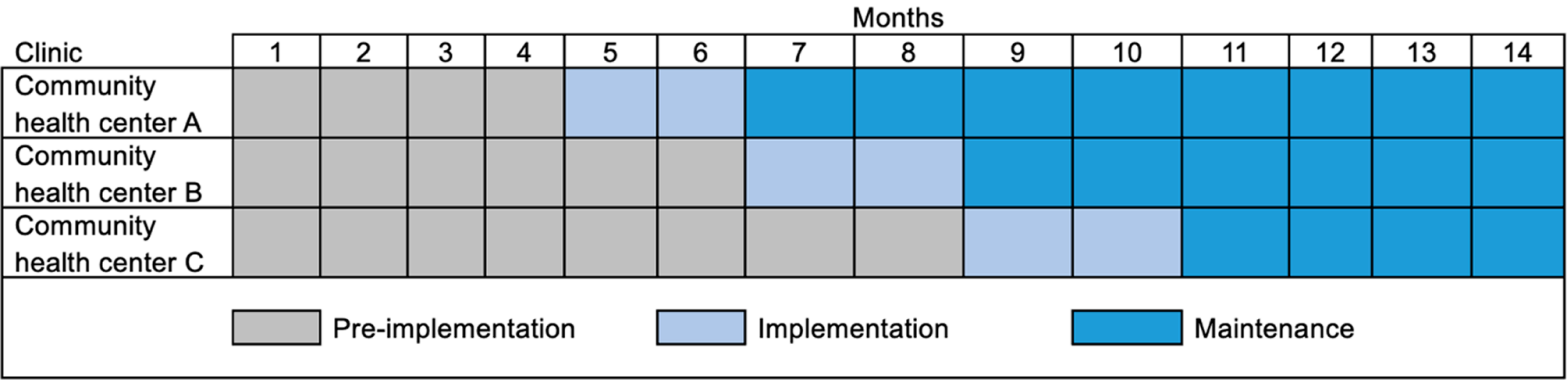
Stepped wedge study design

We will use a stepped-wedge cluster randomized controlled pilot trial design to evaluate the feasibility of an implementation strategy designed to increase adoption of chiropractic care for LBP. A stepped-wedge design allows each clinic to receive the implementation strategy, with the pre-implementation period acting as the control [34]. Three primary care clinics at CHCs will be randomized to receive the implementation strategy first, second, or third over a fourteen-month study period as shown in Fig. 1. Clinic randomization will be overseen by a biostatistician. At our first clinic, we will have a four-month pre-implementation period, a two-month implementation deployment period, and a subsequent eight-month follow-up period. We will stagger the start of our implementation strategies, beginning in a new clinic every two months. The study protocol was approved by the Boston University Medical Campus IRB. We will conduct parallel qualitative interviews throughout the study period with PCPs, staff, and DCs to help understand if proposed strategies are perceived as feasible within local CHCs. PCPs, DCs, and administrative staff participating in surveys and in-depth interviews will provide consent via an online survey. A waiver of consent was approved for collection of patient demographic information and PCP orders for LBP from the Electronic Health Record (EHR). Reporting will be consistent with the Standards for Reporting Implementation Studies (STaRI) statement [35]. 

### Study setting

We invited eight CHCs to participate in the pilot study. All invited CHCs were part of Boston HealthNet, a network of CHCs in eastern Massachusetts, and none had an embedded chiropractic care service. We submitted a standardized form to Boston HealthNet that provided information about the study so that CHCs could determine if the project aligned with their priorities and if partnership was feasible. Of the eight CHCs, three were interested in participating, one declined, and four did not respond. One of the three interested CHCs was unable to participate due to staff turnover of their research representative responsible for managing new research projects. One of the other interested sites, NeighborHealth, was split into two sites that provide care in separate buildings and operate like independent practices, i.e., the Adult Medicine and Family Medicine primary care clinics were separate sites. The third site was The Greater Roslindale Medical and Dental Center.

### Patient population

During the fourteen-month study period, we will identify all patients aged 18 or older who have a diagnosis of LBP documented in the EHR by their PCP at a participating clinic. This will include acute or chronic LBP with or without leg pain using appropriate ICD codes (e.g., M54.5, M54.1, M54.16, M54.41, M54.42, M54.31, M54.32, M48.06, M48.07, M51.16, M51.17, M99.83, S33.5) [36]. We will exclude patients with a red flag diagnosis suggesting serious pathology may be the cause of LBP (e.g., cauda equina syndrome, cancer, spine infection or fracture). We will also exclude patients who had a primary care visit for LBP within the prior 90 days. Thus, patients with LBP may enter our study more than once if they have a new episode of LBP with 90 days between primary care visits.

### PCPs, CHC staff, and DCs

PCPs with various clinical training (doctor of medicine [MD], doctor of osteopathy [DO], nurse practitioner [NP], or physician assistant [PA]) who practice at the CHCs will be invited to participate in our study through completing surveys or in-depth interviews. Administrative staff from participating CHCs, and community-based DCs will also be invited to participate in in-depth interviews.

### Clinical intervention

We designate ‘chiropractic care’ as the evidence-based treatment for LBP to be adopted by the primary care clinics [37]. Chiropractic care typically combines self-care approaches (e.g., exercise or stretching instruction) and evidence-based nonpharmacologic treatments (e.g., spinal manipulation, massage) [38]. As described above, this approach is consistent with current LBP clinical practice guidelines [7, 9, 10, 16, 17]. 

### Implementation strategy

**Fig. 2:**
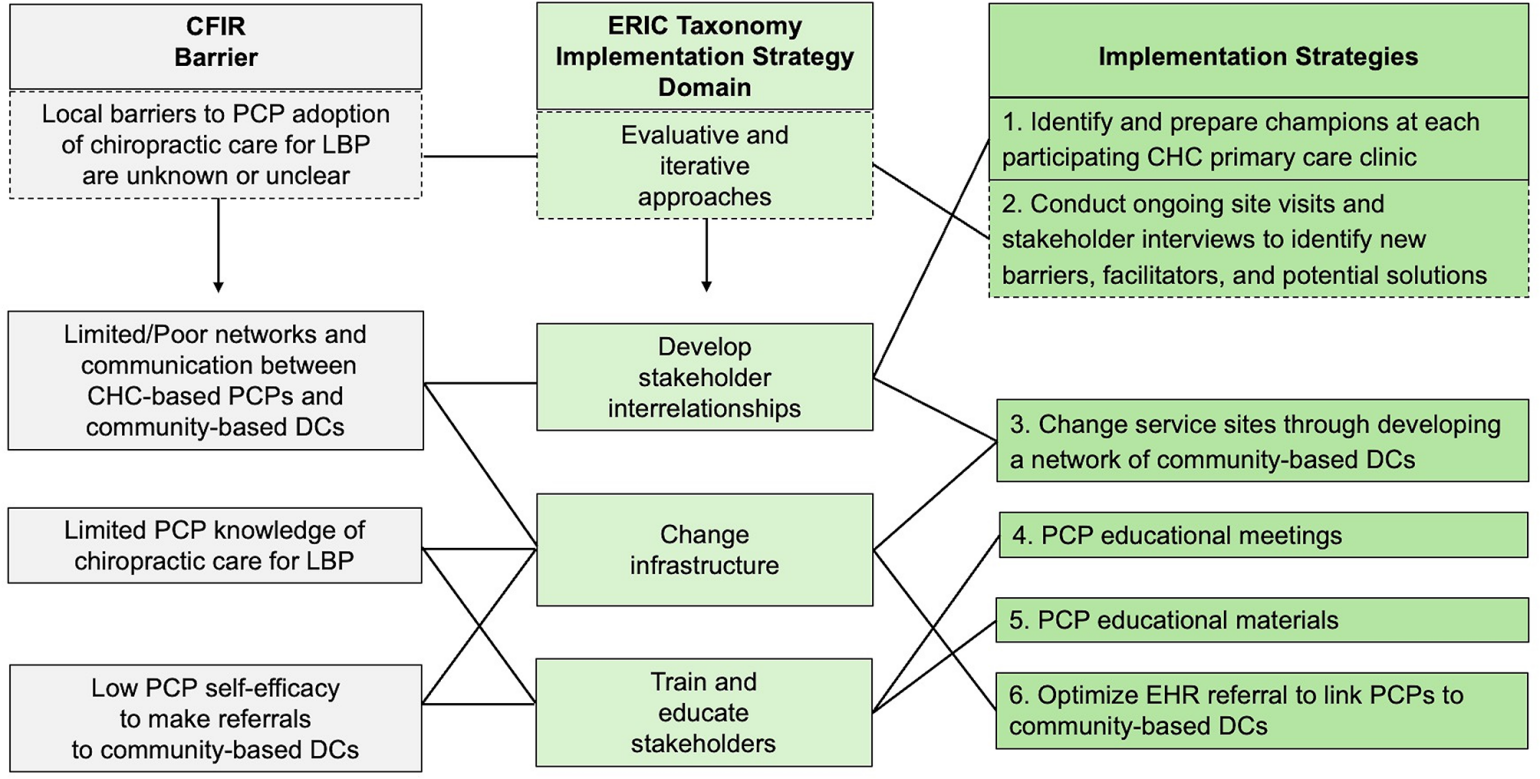
Mapping of known barriers to preliminary multi-level mplementation strategy. Diagram shows the expected barriers using language from the consolidated framework for implementation research (CFIR), relevant implementation strategy domains from the ERIC Taxonomy, and the specific implementation strategies selected for pilot study. We identified six distinct strategies which are further specified in Table 1

Implementation strategies intend to increase the rate at which evidence-based clinical interventions (i.e., chiropractic care) become part of routine management of a given health condition (i.e., LBP) [37, 39]. Implementation strategies are thought to be most effective if they target known barriers and facilitators and are tailored to the local context (i.e., CHC primary care clinics). Known determinants of implementation from the Consolidated Framework for Implementation Research (CFIR) [40, 41] were considered during our prior studies of PCPs working in local primary care clinics [32, 42]. Additional engagement of stakeholders at the three participating sites will help us to understand whether strategies are feasible and how to further tailor strategies to meet local needs. Using the Expert Recommendations for Implementing Change (ERIC) Taxonomy of implementation strategies [43, 44] as a guide, our multi-level implementation strategy will use six discrete strategies that target known barriers as shown in Fig. 2.

**Table 1:** Specifications of the six components of the multi-level implementation strategy

	Proctor et al. categories for reporting on implementation strategies					
Implementation strategies	Target	Actor	Action	Outcome	Temporality	Dose
1. Identify and prepare champions	Champion	Implementation team	Engage in development of implementation strategies	Identify champion to facilitate rollout of implementation strategy	Pre-implementation	Once monthly, more frequently as needed
2. Assess for readiness and identify barriers and facilitators	PCPs, Staff, DC	Implementation team	Interviews, site visits	Tailor implementation strategy to local needs	Pre-implementation, Follow-up period	*≥* 8 interviews pre- and during/post-implementation
3. Generate list of community-based chiropractors	PCP	Implementation team	Identify and interview local DCs	Generate list of local DCs who meet vetting criteria suggested by PCPs (e.g., accepts Medicaid/Medicare, location of practice)	Pre-implementation, updated based on PCP/patient feedback	Once, updated as needed
4. Educational meetings for PCPs and DCs	PCP, DC	Implementation team	Deliver presentation	Increase knowledge and improve acceptability or appropriateness of chiropractic care for LBP	Implementation period	Once monthly
5. Weekly email to PCPs (educational materials)	PCP	Champion	Send email	Increase knowledge and improve acceptability or appropriateness of chiropractic care for LBP	Implementation period	Once weekly
6. Changes to the electronic health record	PCP	Local IT	Optimize DC referral	Increase ease of referral to chiropractic care for LBP	Implementation period, Follow-up period	Once, updated as needed

The six implementation strategies are operationalized in Table 1 using the seven criteria for reporting of implementation strategies as recommended by Proctor et al. [45]. First, we will identify and prepare at least one study champion at each CHC with a leadership role (e.g., research liaison for CHC, medical director) who will help to tailor and deploy the other implementation strategies. Second, we will assess readiness and identify barriers and facilitators. This will be accomplished through site visits and engaging key stakeholders (PCPs, staff, DCs) during in-depth interviews to identify additional local barriers and to help guide local tailoring of implementation strategies. Third, we will generate a network of community-based DCs. We will identify DCs using the state licensure database and create a list of practice locations. Chiropractic clinic locations will be verified by contacting DC or clinic staff via email, phone, and/or during site visits. Local DCs will be invited to participate through informal brief conversations and in-depth interviews. Fourth, we will host two educational meetings at each CHC for PCPs and staff to attend. The first educational meeting will include content on the effectiveness and safety of chiropractic care and additional topics identified as important through site visits and interviews (preliminary topics are shown in Table 2). During the second educational meeting, local DCs will be invited to participate as part of a panel on why and how to refer to chiropractic care. Fifth, we will develop and distribute educational materials to PCPs at each CHC clinic. Content from the educational meetings will be re-packaged in six brief educational messages that will be distributed to all PCPs. Sixth, we will work with CHC primary care clinics to improve their referral mechanism, mainly through adding DCs from the network (strategy 3) to the order form in the EHR, i.e., allowing PCPs to select from community-based DCs using information on practice location, whether the practice accepts Medicare/Medicaid, and the languages DCs or their staff speak other than English. We will continue to engage community-based DCs across the study-period to increase referral options for PCPs and their patients.

**Table 2:** Educational modules to inform educational meetings and materials

#	Title	Core message	Key reference
1	What is chiropractic care?	Review the core components of chiropractic care including evaluation and management including spinal manipulation, other manual therapies, exercise instruction.	Beliveau et al. 2017 [15] Hartvigsen et al. 2020 [46]
2	Educational standards	Review educational standards of DCs. Chiropractors are providers who specialize in the management of musculoskeletal conditions, particularly LBP.	CCE NBCE
3	Disparities in access	Review demographic groups with varying access to chiropractic care among Americans with LBP including patients who have recently seen their PCP.	Roseen et al. 2023 [14] Joyce et al. 2024 [12] Heyward et al., 2020 [31]
4	Acute LBP	Chiropractic care is safe and effective for acute LBP and is recommended in clinical practice guidelines for these patients	Paige et al. 2017 [18] Qaseem et al. 2017 [7] Goertz et al., 2018 [25]
5	Chronic LBP	Chiropractic care is safe and effective for chronic LBP and is recommended in clinical practice guidelines for these patients	Rubenstein et al. [20] Qaseem et al., 2017 [7] Goertz et al., 2018 [25]
6	Back-related leg pain	Chiropractic care is safe and effective for subacute and chronic back-related leg pain and recommended in clinical practice guidelines for these patients	Bronfort et al., 2014 [47] Bussieres et al., 2021 [10] Schneider et al., 2019 [48]
7	Treatment duration	Many patients with acute LBP will benefit from a brief trial of chiropractic care (e.g., 8 visits). The optimal dose of spinal manipulation for chronic LBP in a RCT was 12 visits. Some patients with persistent symptoms may benefit from ongoing management although treatment should become less frequent, once every 1–3 months.	Whalen et al. 2022 [24] Haas et al. 2014 [49] Eklund et al. 2018 [50]
8	Safety of chiropractic care	Chiropractic care is generally safe for LBP with mild adverse events being common (e.g., increase in soreness) and serious adverse events (e.g., fracture, disc injury) being rare. Relative safety versus over-the-counter pain medications is a main reason nonpharmacologic treatment is recommended as first-line therapy for LBP in clinical practice guidelines.	Gouveia et al. 2009 [21] Hebert et al. 2015 [22] Qaseem et al. 2017 [7]
9	Reliance on pain medications	Patients who see a chiropractor for LBP, compared to those who see their PCP first, are less than half as likely to receive an opioid in the short or long term	Corcoran et al. 2020 [26] Kazis et al. 2019 [27]
10	Cost-effectiveness of chiropractic care	Nonpharmacologic treatments, including chiropractic care, are consider cost effective for LBP	Andronis et al. 2017 [23] Blanchette et al. 2016 [24]
11	Making referrals	Logistics of making referrals. Discuss barriers and facilitators that patients may experience when trying to access chiropractic care	N/A
12	Local DCs	Review network of local community-based chiropractors	N/A

CCE: Council on Chiropractic Education; NBCE: National Board of Chiropractic Examiners

## Measurement

Below we define measures for the following domains: (1) feasibility outcomes; (2) adoption of chiropractic care and other treatments; and (3) patients and PCP characteristics that may predict adoption of chiropractic care for LBP. All variables are described in Table 3.

**Table 3:**
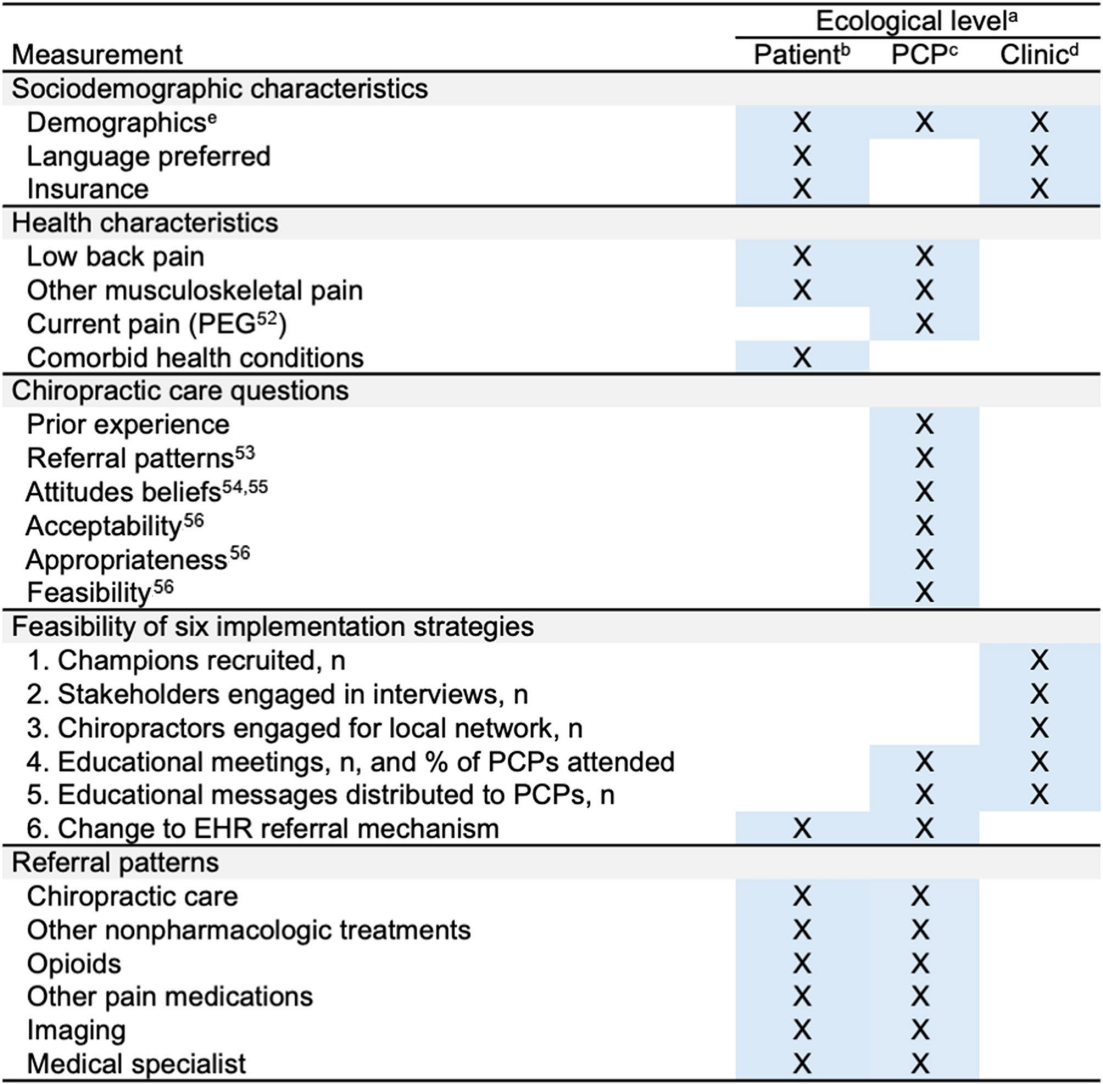
Patient-, PCP-, and clinic- level measurements

^a^Measurements at different ecological levels will include patient-, PCP- and clinic-level measurements

^b^Patient-level characteristics for individuals with low back pain will be measured from electronic record at time of index visit. Referral patterns for patients with LBP are within 21 days of index visit

^c^PCP-level characteristics will be obtained via administrative data and an online survey

^d^Clinic-level characteristics will be provided by clinic champion from administrative data and include aggregate characteristics of patient or provider demographics

^e^Demographic characteristics will include age, sex, race, and ethnicity

### Feasibility outcomes

We will use descriptive statistics to present whether it was feasible to recruit clinics to participate in a fourteen-month study with a 2-month implementation phase. Based on EHR data, we will determine if it is feasible to identify patients with LBP and relevant PCP referrals. Additionally, we will describe the feasibility of completing each of the six implementation strategies. We will report on each of the seven dimensions (e.g., temporality, dose) specified in Table 1, indicating whether it was delivered as intended, if it could not be delivered, or if it occurred with some adaptation to what was pre-specified. Thus, we will describe whether each implementation strategy occurred, e.g., were we able to send out educational materials to all PCPs, and the dose of each, e.g., how many education modules did we send out to PCPs for each CHC.

### Adoption of chiropractic care and other treatments

Adoption of chiropractic care will be measured through PCP referrals to chiropractic care. We will extract this information from the EHR based on orders placed following an index visit with a PCP for LBP. The rate of adoption for a given month will be the proportion of index visits where an order for chiropractic care is placed within 21 days. This period of time is based on a recent study showing that most patients receive at least one order within 21 days of an initial visit to their PCP for acute LBP and that orders after 21 days are uncommon [12]. This study also found that < 5% of participants with acute LBP received an order to chiropractic care within 21 days [12]. Additionally, we will identify other recommendations documented in the EHR by PCPs within 21 days of the index visit. These will include: (1) other evidence-based nonpharmacologic treatments (e.g., acupuncture, massage, physical therapy); (2) prescribed pain medications (e.g., NSAIDs, opioids); (3) referral to imaging (plain x-rays, MRI), or referral to specialists (e.g., sports medicine, orthopedics) who may offer interventional procedures (e.g., epidural injections, surgery).

### Patient and PCP characteristics

For patients with an index visit for LBP during the study period, we will collect information from the EHR on demographic characteristics (e.g., age, gender, race/ethnicity), specific LBP diagnosis (e.g., lumbago, lumbar radiculopathy, lumbar spinal stenosis), comorbid health conditions, and prescription of an opioid or other pain medications in the prior year.

PCPs will be invited to complete an online survey to gather additional information on sociodemographic characteristics (e.g., age, sex, race and ethnicity), pain experiences, attitudes/beliefs regarding chiropractic care, and additional constructs that may predict adoption of chiropractic care. Prior personal experiences of pain and pain treatment may influence PCP management decisions for their patients with LBP. Thus, PCPs will be asked about current or previous personal experiences of musculoskeletal pain including the location(s) and duration of pain. The 3-item PEG Pain Screening Tool will be used to assess their pain on average, how much the pain has interfered with their enjoyment of life, and how much the pain has interfered with their general activity [51]. Each question is scored from 0 to 10 with higher scales indicating worse pain. PCPs will be asked if they have received chiropractic care (yes/no) and, if yes, whether their experience was positive, neutral, or negative. Additionally, we will ask PCPs to self-report patterns of referring to DCs using questions from the National Ambulatory Medical Care Survey [52] including “Have you referred to a chiropractor in the past 12 months?” with participants responding “yes/no” and “How often have you referred your patients to chiropractic care in the past 12 months?” with participants responding “weekly, monthly, quarterly, a few times per year, never”. Attitudes and beliefs towards chiropractic care for LBP will also be captured by adapting questions from previous studies of chiropractic care in primary care [53] and chiropractic experiences among medical students [54]. To understand the acceptability, appropriateness, and feasibility of routine chiropractic care use for LBP among PCPs, we will adapt questionnaires developed by *Weiner et al.*: The Acceptability of Intervention (AIM), Intervention Appropriateness Measure (IAM), and Feasibility of Intervention Measure [55]. Each questionnaire includes four items and a corresponding 5-point Likert scale, allowing responses to range from 1 (strongly disagree) to 5 (strongly agree) for each item. Higher scores indicate greater acceptability, appropriateness, and feasibility [55]. 

### Data analysis

#### Feasibility outcomes

We will use descriptive statistics to summarize the number of months each clinic participated in the study, the number of patients with LBP per month at each site, and the number of each type of PCP recommendation for LBP treatment documented in the EHR. We will describe the provider and patient characteristics using descriptive statistics. Additionally, we will describe whether each of the six implementation strategies was completed for the three participating primary care clinics. We will report on each of the seven dimensions (e.g., temporality, dose) for the six implementation strategies specified in Table 1 as described above.

#### Adoption of chiropractic care and other LBP treatments

Adoption of chiropractic care and other LBP treatments will be presented as the proportion of index visits each month receiving a particular PCP order type. We anticipate that adoption of chiropractic care will be our primary outcome in a future large fully powered cluster randomized trial with other PCP treatment recommendations being important secondary outcomes. The adoption of chiropractic care or other LBP treatments will be illustrated in figures showing the prevalence of orders over the study period.

In the analysis of adoption of chiropractic care, we will create a dichotomous measure “chiropractic referral” (yes/no). We will compare chiropractic referrals before the deployment of the implementation strategy and after deployment begins (i.e., pre-implementation vs. [deployment + follow-up]). The proportion of LBP patients receiving a chiropractic referral for these two time periods will be calculated. The numerator will be those with a chiropractic referral, defined as those LBP patients who received a referral, and the denominator will be all eligible LBP patients. We will use random effects logistic regression adjusting for fixed and random effects at the clinic level and a fixed effect for time of deployment of implementation strategy to estimate the change in the proportion of CHC patients with LBP who are referred to chiropractic care. While main analyses will evaluate initial referrals (i.e., those placed within 21 days of index visit), we will also explore delayed referrals in sensitivity analyses (i.e., referrals placed 22 to 89 days following index visit).

The above analyses will then be repeated to evaluate changes in other LBP treatments over the study period. We will compare the proportion of LBP patients who are referred to other nonpharmacologic treatments (e.g., PT, acupuncture), prescribed pain medications (e.g., opioids), referral to imaging (e.g., MRI), or referral to medical specialist (e.g., sports medicine, orthopedics). We will also identify interventional procedures for LBP (e.g., epidural injections, surgery) documented in the EHR during the study period.

#### Predictors of LBP treatment

Additional analyses will identify predictors of the dichotomous outcome of ‘chiropractic referral’ using logistic regression with PCP and patient factors serving as predictor variables. This analysis will be repeated with other PCP recommendations (e.g., opioid prescription) as outcome for model. This will provide preliminary information on whether PCP and patient characteristics that predict referral to chiropractic care also predict referrals to other guideline concordant or discordant treatments. We will use exploratory analysis (multi-level predictors of chiropractic referral) to explore which baseline characteristics predict chiropractic referral for LBP using logistic regression. We will build a general model using the ‘chiropractic referral’ outcome as the dependent variable with patient and provider characteristics as independent variables.

#### Qualitative interview procedures, data and analyses

Interview guides informed by the consolidated framework for implementation research (CFIR) were updated from prior studies of implementation barriers and facilitators [32, 41, 56]. Interviews will be focused on identifying barriers and facilitators from stakeholders during the pre-implementation, implementation, and follow-up phases. Tailoring of implementation strategies will be informed by interviews during pre-implementation and implementation phase. Understanding whether these strategies were feasible and helpful in addressing barriers will be explored during interviews of intervention phase and during follow-up phase.

Interviews will last 30 to 60 min. Following the interview, study staff will generate a transcript verbatim and an initial memo to highlight key barriers, facilitators, and potential implications for pilot study. Initial memos will be developed immediately following the interview and discussed at weekly team meetings to help inform and track tailoring or adaptions to the implementation strategies during the course of the project [57]. The research team will then take a multi-step approach to analyze interview data. Each transcript will be coded independently by two coders. After four transcripts are double coded, the two coders will meet with a medical anthropologist (LL) to review coding process, clarify use of codes, and discuss any changes to the codebook. Throughout this process coding will be discussed at weekly meetings. The analysis of coded transcripts will be completed by team members with implementation science training and experience (EJR, LL). Codes and coded text will be reviewed to ensure that they were linked to the appropriate CFIR construct. A senior implementation scientist (AB) will then review the resulting categories to ensure that barriers are mapped appropriately to CFIR constructs. The main analyst will then outline the main points for each category within the matrix along with illustrative quotes from interviews.

## Discussion

We are unaware of any prior prospective trials that have explored a multi-level implementation strategy designed to increase the adoption of chiropractic care for LBP in the CHC primary care setting. We will use two frameworks from the field of implementation science to guide the design and conduct of our study which will address this important knowledge gap. First, we will use CFIR to identify barriers to adoption of chiropractic care for LBP in CHC primary care clinics. Second, will use the ERIC Taxonomy of Implementation Strategies to develop a tailored multi-level implementation strategy that is designed to address the identified barriers [41, 43, 44]. Our study will serve as a critical step towards understanding how healthcare settings can be redesigned to better incorporate nonpharmacologic treatments such as chiropractic care. The proposed work will set the stage for a future large implementation study to evaluate the effectiveness of our multi-level implementation strategy to increase the adoption of chiropractic care for LBP within the CHC context. Further development and use of such implementation strategies by CHCs is needed to address the lack of access to evidence-based spine care services in marginalized populations.

While prior work from Canada [58–60] and the United States [61] has shown that offering chiropractic care within CHC primary care clinics is feasible, few CHCs offer embedded chiropractic care. Linking CHC PCPs to community-based DCs has the appeal of rapidly expanding access to chiropractic care to meet the demand as has been observed in the community-care initiative of the Veterans Health Administration [62]. Furthermore, this may be a necessary step towards growing the DC workforce that is accessible to underserved civilians who receive care in CHCs.

Our multi-level implementation strategy includes 6 distinct implementation strategies designed to increase PCP knowledge of chiropractic care for LBP, develop connections between PCPs and community-based DCs, and reduce logistical challenges in the referral process by optimizing the referral in the EHR. This approach is practical and addresses prominent themes from prior interviews of PCPs on why they refer infrequently to chiropractic care and other nonpharmacologic treatments [32]. However, we anticipate once these barriers are reduced, additional strategies may be needed to address remaining barriers and further increase and sustain adoption. For example, strategies that directly target patients with LBP may help promote patient-initiated conversations about chiropractic care as a treatment option with their PCP. While we initially intended to incorporate patient-facing brochures and other printed educational materials, feedback from study champions during the planning phase indicated that PCPs may not use printed resources due to a lack of time. Through our interviews, we will explore the ideal method for delivering educational materials directly to patients. This could include embedding materials as part of the after-visit summary or sharing electronic materials through the online patient portal (e.g., MyChart in Epic).

Our study has important limitations. First, we will include only three CHC primary care clinics and will be underpowered to assess the effectiveness of our multi-level implementation strategy for adoption outcomes in this pilot study. Second, all participating CHC primary care clinics are within Suffolk County, an urban area in eastern Massachusetts. Thus, we anticipate a future large adequately powered trial would include a larger number of CHCs from suburban and rural areas. Third, only four of eight Boston HealthNet CHCs responded to our initial invitation to participate in the pilot study. Thus, we anticipate we will need to work with our CHC champions to better understand CHC priorities and competing demands in preparing for a future study that will recruit additional CHC primary care clinics.

Our plan for dissemination of findings includes several reports. First, the main outcomes and analyses described in this protocol will be published in a timely manner following trial completion. Second, we anticipate publishing at least one additional manuscript that summarizes themes of qualitative interviews on barriers and facilitators of chiropractic care for LBP. Third, we will develop a report on patient and PCP characteristics that predict adoption of chiropractic care in primary care clinics. We posit that the size of the local DC workforce and the geographic location of chiropractic clinics may also be associated with adoption of chiropractic care in primary care clinics. Thus, a fourth planned manuscript will use geospatial analysis to explore access to chiropractic care in neighborhoods of Boston that are near participating CHCs. In addition to our scholarly articles, we will work with the participating CHCs and our advisory board (described below) to explore the best method to widely communicate lessons learned from this work [63]. Study data will be made available on formal request to the principal investigator and following completion of a data use agreement.

After completing the implementation phase at all three sites, we will form an advisory board to provide feedback on developed implementation strategies and to provide additional guidance on how to scale or transfer strategies in additional CHCs or other healthcare settings. Additional strategies may also be needed in future implementation efforts. We will seek guidance from the advisory board on any barriers that emerge during our ongoing site visits and stakeholder interviews. The use of advisory committees has been part of the approach to implementing chiropractic care in the Veterans Health Administration [64]. The advisory board will be made up of local and national experts including leaders from other CHCs who may serve as sites for future trials. The results from a future large trial could inform policy makers on the need to sustain this approach to help reduce PCP workload, reduce overall spine care costs (reductions in use of imaging or procedures), and increase treatment safety (reduce opioid use).

## Electronic supplementary material

Below is the link to the electronic supplementary material.

Supplementary Material 1

Supplementary Material 2

## Data Availability

No datasets were generated or analysed during the current study.

## Abbreviations

CFIR: Consolidated Framework for Implementation Research
CHC: Community health center
DC: Doctor of Chiropractic
EHR: Electronic Health Record
ERIC: The Expert Recommendations for Implementing Change
LBP: Low back pain
NSAID: Non-steroidal anti-inflammatory drug
PCP: Primary care provider
